# Butyrate and hexanoate-enriched triglycerides increase postprandrial systemic butyrate and hexanoate in men with overweight/obesity: A double-blind placebo-controlled randomized crossover trial

**DOI:** 10.3389/fnut.2022.1066950

**Published:** 2023-01-04

**Authors:** Thirza van Deuren, Lotte Smolders, Anita Hartog, Freek G. Bouwman, Jens J. Holst, Koen Venema, Ellen E. Blaak, Emanuel E. Canfora

**Affiliations:** ^1^Department of Human Biology, School for Nutrition and Translational Research in Metabolism (NUTRIM), Maastricht University Medical Center+, Maastricht, Netherlands; ^2^AAK, Department of Biotechnology and Nutrition, AAK Netherlands BV, Zaandijk, Netherlands; ^3^Novo Nordisk Foundation Center for Basic Metabolic Research, University of Copenhagen, Copenhagen, Denmark; ^4^Department of Biomedical Sciences, University of Copenhagen, Copenhagen, Denmark; ^5^Centre for Healthy Eating and Food Innovation, Maastricht University, Venlo, Netherlands

**Keywords:** butyrate, hexanoate, obesity, metabolic health, short chain fatty acids

## Abstract

**Background:**

Short chain fatty acids (SCFA) are increasingly recognized for their potential ability to alleviate obesity-associated chronic low-grade inflammation and disturbed energy homeostasis. Evidence suggests that an increase in circulating SCFA might be necessary to induce beneficial alterations in energy metabolism.

**Objective:**

To compare the bioaccessibility of two different SCFA-enriched triglycerides: Akovita SCT (butyrate and hexanoate esterified with long chain fatty acids) and tributyrin/caproin (solely butyrate and hexanoate) and investigate whether the SCFA from orally administrated Akovita SCT reach the circulation and affect postprandial metabolism in men with overweight/obesity.

**Methods:**

The site, speed, and amount of SCFA release from Akovita SCT and tributyrin/caproin were assessed in a validated *In vitro* Model of the stomach and small intestine (TIM-1). Subsequently, a double-blind placebo-controlled randomized crossover study was conducted at Maastricht University with fourteen men with overweight/obesity (BMI 25–35 kg/m^2^) of which twelve men finished all testdays and were included for analysis. The participants received a liquid high fat mixed meal test containing either a low (650 mg), medium (1,325 mg), or high dose (2,000 mg) of Akovita SCT or a placebo (sunflower oil) in randomized order. Blood was sampled at baseline and after ingestion for 6 h for the primary outcome plasma butyrate and hexanoate concentration. Secondary outcomes included hydrogen breath, appetite, gastrointestinal complaints, circulating glucagon-like peptide 1, free fatty acids, glucose, triglycerides, insulin, and cytokines concentrations.

**Results:**

In TIM-1, tributyrin/caproin was rapidly cleaved in the gastric compartment whereas the release of SCFA from Akovita SCT occurred predominantly in the small intestine. *In vivo*, all doses were well-tolerated. The medium dose increased (*P* < 0.05) and the high dose tended to increase (*P* < 0.10) postprandial circulating butyrate and both doses increased circulating hexanoate (*P* < 0.05) compared to placebo. Nevertheless, Akovita SCT supplementation did not affect any secondary outcomes compared to placebo.

**Conclusion:**

Esterifying SCFA-enriched triglycerides with long chain fatty acids delayed SCFA release from the glycerol backbone. Akovita SCT increased postprandial circulating butyrate and hexanoate without changing metabolic parameters in men with overweight/obesity. Future randomized clinical trials should investigate whether long-term Akovita SCT supplementation can aid in the treatment or prevention of metabolic disorders.

**Clinical trial registration:**

www.ClinicalTrials.gov, identifier: NCT04662411.

## 1. Introduction

Obesity is a major public health concern and novel therapeutic strategies are urgently required to prevent and treat obesity and associated metabolic disorders. This non-communicable disease is characterized by chronic low-grade inflammation and a disturbed substrate and energy metabolism, which may eventually impair insulin signaling and result in insulin resistance (1, 2). Based on data derived from a wealth of animal studies and limited human data, short chain fatty acids (SCFA) may provide a valuable strategy to combat obesity and obesity-related complications. SCFA, of which acetate, propionate, and butyrate are most abundant, are microbial metabolites mainly produced from the fermentation of indigestible carbohydrates in the colon. These SCFA are often put forward as the gateway between the gut microbiota and host metabolism and are hypothesized to be the key explanation for the beneficial metabolic effects associated with dietary fiber consumption (3, 4). SCFA can bind to G-protein coupled receptors (GPR) including GPR41 and GPR43, which are present in several metabolically active tissues including the intestine, liver, and adipose tissue (5–8). By binding to these GPR, SCFA may stimulate the production of gut-derived hormones such as glucagon-like peptide (GLP-1) and thereby influence appetite and weight regulation (9–11). This GLP-1 inducing effect may also partly explain its beneficial on insulin sensitivity. GLP-1 increases blood volume and blood flow of the muscular microvasculature, thereby (indirectly) increasing insulin delivery to the muscle (12, 13). Moreover, when primary myocytes from individuals with obesity were incubated with GLP-1, increased glucose uptake was observed (14).

Although all three SCFA have been implicated in improvements in metabolic health (4), the 4-carbon SCFA butyrate has received particular attention due to its pronounced observed beneficial effects on weight status and insulin sensitivity in obese and diabetic mouse models (15, 16). *In vitro* studies using pancreatic cell lines propose that butyrate, besides indirectly increasing insulin sensitivity via GLP-1 production, may also have an acute insulinotropic effect (17, 18). Next to binding to GPR, butyrate can also act as a histone deacetylase (HDAC) inhibitor and thereby has to ability to modulate gene expression, which may, among others, contribute to its anti-inflammatory properties (19, 20). Additionally, butyrate may epigenetically upregulate the expression of the μ-opioid receptor (21). This receptor is involved in the hedonic and reward-related aspects of food intake, and its observed reduced expression in individuals with obesity is thought to play a role in excessive eating behavior (22). Animal studies have demonstrated that butyrate supplementation may prevent diet-induced weight gain (10, 23–31), positively affect systemic inflammatory markers profiles (25, 29, 32–34), increase the production of anorexigenic hormones including GLP-1 (10, 11), shift substrate metabolism from carbohydrate to lipid utilization (26, 27), and improve insulin sensitivity and glucose tolerance (26–30, 35, 36).

Nevertheless, at present, data are predominantly derived from animal studies and *in vitro* studies using supraphysiological concentrations whereas our understanding of the effects of butyrate on human metabolism remains limited. In a large cohort of normoglycemic individuals, mendelian randomization analyses have linked the abundance of several butyrate-producing microbial strains to an improved postprandial insulin response (37). Interestingly, circulating butyrate was inversely associated with body mass index (BMI), indicating systemic butyrate levels may be decreased in individuals with obesity (38). However, data from well-controlled human clinical trials are limited and the outcome may depend on metabolic phenotype (4, 15). For example, 4 weeks of daily sodium butyrate (4 grams) supplementation only improved insulin sensitivity in lean men but not in men with metabolic syndrome (39). Additionally many long-term butyrate-focused interventions aimed at improving metabolic health are not placebo controlled (39–44) and human studies investigating the acute effect on GLP-1 release and substrate and energy metabolism in response to butyrate are lacking (15).

Although butyrate has received a lot a scientific attention, the 6-carbon SCFA hexanoate, also known as caproate, is also produced by several anaerobic bacteria colonizing the gut (45). This SCFA is produced in smaller quantities than the three main SCFA, but *in vitro* work suggests that hexanoate may have beneficial effects on lipid metabolism and insulin sensitivity in the liver (46, 47). Hexanoate can also bind to GPR41 and GPR43, albeit with less potency than butyrate, and both receptors have been implicated in anti-inflammatory processes (6, 48). In obese mice, 8 weeks of hexanoate supplementation prevented diet-induced hepatic glycogen reduction (49). Nevertheless, hexanoate remains a scarcely studied metabolite and human clinical studies with hexanoate have not been reported so far. Based on the properties of both SCFA, combining butyrate and hexanoate may be a promising approach to combat obesity-associated chronic low-grade inflammation and tissue-specific metabolic dysfunctions. Nevertheless, sodium butyrate (which is most commonly used in animal and human studies) is known for its unpleasant flavor and odor, which calls for a more edible and palatable version for future (clinical) use. By attaching the SCFA to a glycerol backbone, esterified with LCFA, an easy-to-use oil that masks butyrate's rancid-smell can be manufactured for daily consumption. Although tributyrin/tricaproin, triglycerides consisting of three SCFA, may have an increased bioavailability compared to sodium butyrate, tributyrin may induce side-effects including nausea and headache. Tributyrin, similar to sodium butyrate, still has some unfavorable sensory attributes, although to a lesser extent (50, 51).

By incorporating SCFA into triglycerides, the absorption and/or release of SCFA in the gastrointestinal tract may also be altered compared to sodium butyrate. A chronic, more steady supply of butyrate to the circulation may be necessary to induce beneficial metabolic effects. SCFA-enriched triglycerides need to be cleaved by gastric and pancreatic lipase in the stomach and small intestine, respectively, to release the free fatty acids (FFA) that can be taken up by enterocytes. In contrast, sodium butyrate is already in its free form and, consequently, does not require cleavage and can be absorbed earlier in the gastrointestinal tract. The location of SCFA absorption may affect the amount of SCFA reaching the circulation. Increasing circulating SCFA levels may be of specific importance since previously performed acute colonic SCFA infusion studies within our department showed that an increase in circulating acetate was associated with changes in metabolic health parameters including beneficial effects on human substrate and energy metabolism (52, 53). Furthermore, another recent acute study reported that elevated fasting plasma butyrate concentrations after supplementing long-chain inulin combined with resistant starch in healthy men, coincided with beneficial metabolic effects including increased energy expenditure, carbohydrate oxidation, circulating peptide YY levels, and an improved postprandial glucose response (54). In accordance, human cross-sectional data showed that circulating, but not fecal, fasting butyrate concentrations were associated with increased circulating GLP-1 as well as lowered fasting glucose and FFA levels. Altogether, these results suggest that increased SCFA levels in the systemic circulation may elicit beneficial metabolic health effects. Nevertheless, evidence suggests that orally ingested butyrate is mainly metabolized in the intestinal tract and liver and, as a consequence of extensive splanchnic extraction, does not or only reaches the circulation in very small proportions (55–58). To illustrate, a kinetics study using isotope tracing in healthy adults demonstrated that only 2% of the ingested 495 mg of ^13^C-labeled sodium butyrate ended up in the circulation (58).

Hence, this study aimed to identify whether an easily consumable butyrate and hexanoate-enriched sunflower oil (Akovita SCT) would have the ability to increase circulating butyrate and hexanoate levels and improve postprandial metabolites, hormones, and inflammatory profile. For this purpose, we first tested the digestibility and release (bioaccessibility) of butyrate and hexanoate from the glycerol backbone in the validated TNO (a Dutch institute for applied scientific research) *In vitro* Model of the stomach and small intestine (TIM-1). Subsequently, we investigated the effects of acute intake of four different concentrations of butyrate and hexanoate in Akovita SCT on our primary outcome parameter, plasma butyrate and hexanoate availability, and on several secondary outcomes including circulating metabolites (glucose, triglycerides, and FFA), hormones (insulin and glucagon-like peptide-1 (GLP-1)), cytokines (tumor necrosis factor alpha (TNF-α), interleukin (IL-6), IL-8, IL-10, and interferon gamma (IFN-γ)), appetite, and hydrogen (H_2_) in breath, during postprandial conditions in men with overweight/obesity. In the *in vitro* study, we hypothesized that the release and uptake of butyrate and hexanoate from Akovita SCT may differ from that of tributyrin/tricaproin. Additionally, *in vivo* we hypothesized that Akovita SCT would dose-dependently increase butyrate and hexanoate concentrations in the systemic circulation and induce beneficial effects on postprandial energy and substrate metabolism.

## 2. Material and methods

### 2.1. *In vitro* study in TIM-1

Before the start of the human study, the digestibility (lipid bioaccessibility) and absorption of the investigational product (Akovita SCT, AAK, Sweden) were assessed in TIM-1 (Supplementary Figure 1), a multicompartmental dynamic *in vitro* model of the upper gastrointestinal tract (59). Lipid bioaccessibility is defined as the release of FFA from a meal in the intestinal lumen and thus the amount that is available for absorption. Bioaccessibility is the first limiting factor for lipid bioavailability, which refers to the ability of a compound to reach the systemic circulation (60, 61). Bioaccessibility (%) of numerous food products have been validated in TIM-1 and these results correlate well with *in vivo* human studies (62, 63). To illustrate, TIM-1 lipid bioaccessibility after administration of a stearic and oleic acid-rich blend was significantly and positively correlated to human serum triglyceride levels in response to the same fat blend (63). In the present study, bioaccessibility was defined as the percentage of SCFA release from the investigational products in TIM-1. In the TIM-1 model, physiological conditions such as temperature, pH, peristalsis, enzyme and bile concentrations of healthy adults were simulated to investigate the kinetics (speed and site of SCFA release and absorption) of butyrate and hexanoate-enriched triglycerides during transit through the gastrointestinal tract. To evaluate this thoroughly, experiments were performed both in the gastric compartment of TIM-1 (focusing on gastric digestion and release), as well as in the complete TIM-1 model (focusing on the release and absorption in the small intestinal compartments). All experiments were performed in duplicate and conducted according to protocol as previously described (63).

Investigational products were mixed within a low-fat milk matrix to maximize solubility of the triglycerides and to simulate intake with a meal. If experiments were conducted with the gastric compartment alone, the model ran for 3 h and samples of gastric efflux (Supplementary Figure 1B) were taken every hour. Experiments with the complete TIM-1 model (gastric and small intestine) were terminated after 5 h and samples were taken every hour from the filtrates collected from the jejunum compartment (Supplementary Figures 1M, N, right), the ileum compartment (Supplementary Figures 1M, N, right), and from the ileal efflux (Supplementary Figure 1H). The ileal efflux provided an estimate of the amount that would be delivered to the large intestine (Supplementary Figure 1H). In this 5-h time-frame, ~80–85% of the meal has transited through the small intestine. After the experiments, a sample was taken from the residue to determine how much of the product still remained in the model and to calculate a mass balance. All collected samples were analyzed for butyrate and hexanoate content by ion-chromatography (Brightlabs, Venlo, the Netherlands) to determine cumulative bioaccessibility.

The following products were studied: 5 g of (a) butyrate and hexanoate fatty acids linked to glycerol as a triglyceride (containing 71% butyrate and 29% hexanoate), herein referred to as tributyrin/caproin) which was compared to (b) butyrate and hexanoate fatty acids that were esterified to glycerol together with long chain fatty acids (LCFA): palm stearin and shea olein (containing 4.7% butyrate and 1.9% hexanoate, herein referred to as Akovita SCT). The Akovita SCT tested is composed of a mixture of different triglyceride structures (0, 1, 2, or 3 SCFA esterified with, respectively, 3, 2, 1, 0, LCFA moieties to the glycerol backbone).

### 2.2. Study population

At Maastricht University, fourteen Caucasian men aged between 40 and 70 years without type 2 diabetes (fasting plasma glucose level <7.1 mmol/L) and a body mass index (BMI) between 25 and 35 kg/m^2^, living in proximity of Maastricht (NL) were recruited via a volunteer database and online media advertisement. Since this concerned a first proof-of-principle study only men were included in this study. It is evident that a next step would be to study whether the observed results are also valid in females. Before study inclusion, participants were screened to determine compliance with all the inclusion and exclusion criteria. Men were considered eligible for the study if their blood pressure was within normal range (systolic blood pressure 100–140 mmHg, diastolic blood pressure 60–90 mmHg) and if their weight was stable for at least the last 3 months before participation. Exclusion criteria included: fasting plasma glucose ≥7.1 mmol/L, gastroenterological diseases, (a history of) diabetes mellitus, a history of major abdominal surgery, cardiovascular diseases, cancer, liver or kidney malfunction, disease with a life expectancy of <5 y, alcohol or drug abuse, excessive smoking (>20 cigarettes per day), and intensive exercise training (>3 h/week). Furthermore, individuals were excluded from participation if they followed a hypocaloric, vegan, or vegetarian diet, used laxatives, prebiotics, probiotics, or antibiotics for 3 months prior to the study or during the study or if they were using any medication that affects glucose, fat metabolism, and inflammation (including, among others, β-blockers, corticosteroids, and cholesterol-lowering medication). During a medical screening, eligibility was evaluated by conducting anthropometric measurements and assessing medical history. Additionally, a blood sample was drawn in fasted state to confirm normoglycemia and proper kidney and liver functioning of all included participants. This study was reviewed and approved by the Medical Ethics Committee of Maastricht University Medical Center+ (MUMC+), performed in accordance with the Declaration of Helsinki and monitored by the Clinical Trial Center Maastricht (CTCM, Maastricht, NL). Prior to obtaining medical information or performing any type of measurements on the participant, all individuals signed a written informed consent. The trial is registered on ClinicalTrials.gov with the number NCT04662411.

### 2.3. Study design

**Figure 1:**
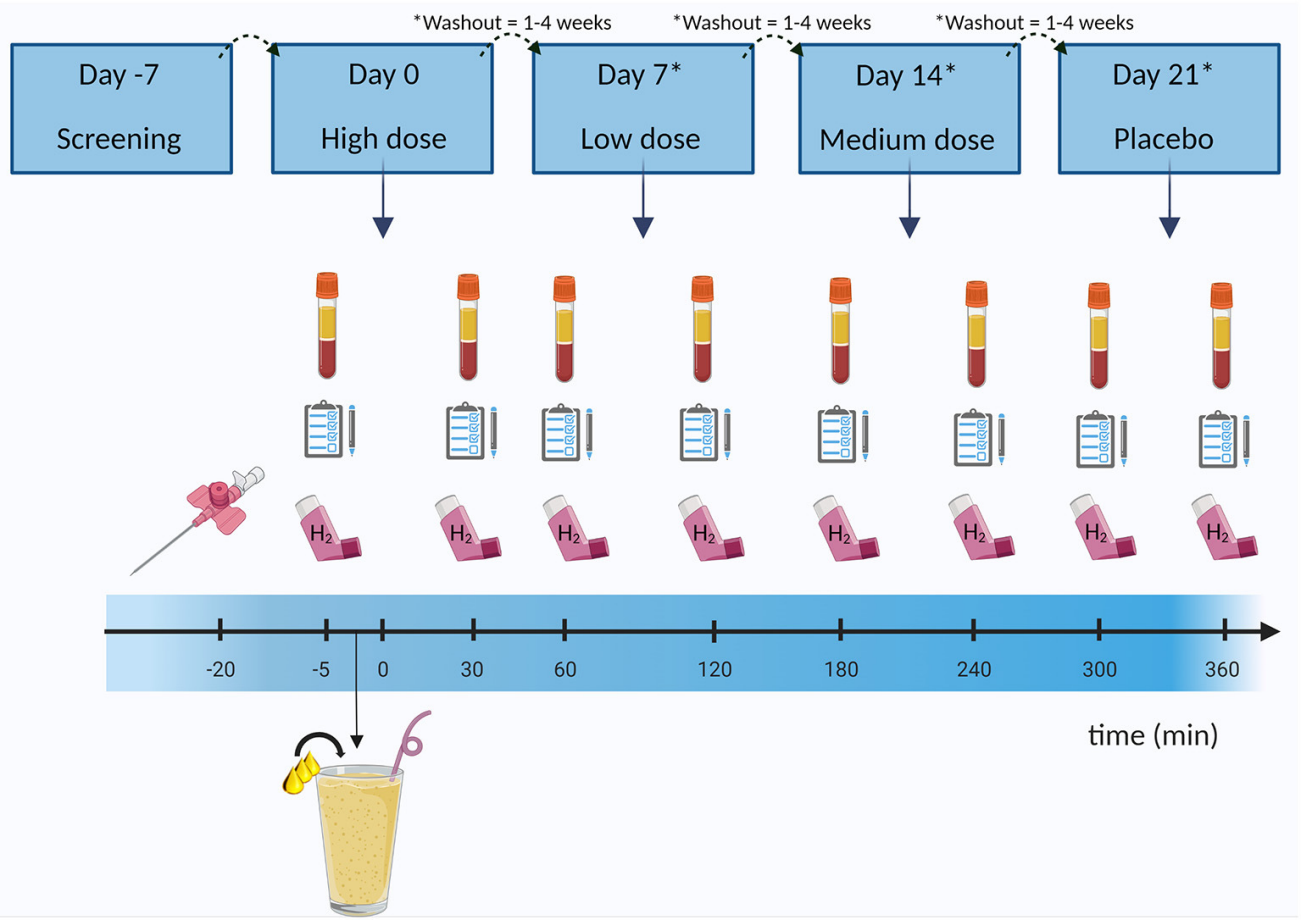
Example of study design and clinical investigation day. After participants were considered eligible for the study, they came in for four clinical investigation days in which they received a liquid high fat mixed meal (milkshake) containing either the placebo (sunflower oil), or a low, medium, or high dose of butyrate and hexanoate-enriched triglycerides (order in which the doses were received was randomized). Blood was sampled at baseline and after ingestion for 6 h. Additionally, hydrogen in breath was assessed by a gastrolyzer and questionnaires were taken to assess gastrointestinal complaints and subjective appetite. Between each clinical investigation days there was a minimum of 1 week (and a maximum of 4 weeks) washout period. H_2_, hydrogen. Created with BioRender.com.

In this double-blind, placebo-controlled, randomized, crossover study, subjects were investigated for four clinical investigation days (CIDs), on which they received an edible oil containing either a low, medium, or high concentration of butyrate and hexanoate-enriched triglycerides (Akovita SCT) or a placebo oil without any SCFA (Fritex high oleic sunflower oil from AAK, Sweden) served as part of a liquid high fat mixed meal (HFMM) (Figure 1). The participants were randomly assigned to the order in which they received these oils by an independent researcher using a computer-generated randomization plan. The order was blinded for investigators as well as for the participants. Randomization was performed for each participant with a sequence ranging from number 1 to 4, representing the different doses (in which each number remained unique within a block/participant, resulting in 24 sequence possibilities). The independent researcher blinded the samples and named the bottles of oil according to the order of CID: 1, 2, 3, and 4. In this way, the researcher knew which bottle of oil to get without knowing the dose that was provided that day.

To prevent any potential carry-over effects from a prior CID, a wash-out period of at least 1 week (with a maximum of 4 weeks) between CIDs was maintained. The evening before each CID, subjects consumed a low fiber standardized meal: “Aviko maaltijdpannetje malse kipfilet” (2.5 g fat, 11.5 g carbohydrates, 4.5 g protein per 100 g of the product). On the CID itself, the participants came in after an overnight fast (≥10 h) and a canula was inserted in an antecubital vein in the elbow to allow continuous venous blood sampling. To ensure any inflammatory responses caused by the needle insertion were stabilized, the baseline (fasted) blood draw was performed 20 min after the canula placement. Participants also filled in a Visual Analog Scale (VAS) for hunger and appetite and a Gastrointestinal Symptom Rating Scale (GSRS) questionnaire to collect gastrointestinal complaints. By filling in a VAS, a participant gives an indication of their subjective feelings toward hunger, fullness, satiety, and the desire to eat by marking on a line from 0 to 100 mm, in which e.g., the left side reflects no desire to eat and the right side reflects extreme desire to eat, to best match to their current state/subjective feeling. In the GSRS, participants were asked to rate the presence any nausea, abdominal pain, abdominal discomfort, bloating, burping/regurgitation, and flatulence on a scale of 0 (not at all) to 5 (a lot). Participants were also able to elaborate on any additional comments concerning gastrointestinal discomfort such as diarrhea or obstipation by the means of an open question. A breath analysis was performed by using a hand-held Bedfont EC60 Gastrolyzer (Rochester, UK) to evaluate the presence of hydrogen in the breath, which is an indicator for global microbial fermentation activity. Hydrogen breath was measured because it was hypothesized that part of the ingested butyrate and hexanoate may reach the microbiota in the ileum and/or colon and subsequently affect fermentative microbial activity e.g., through cross-feeding pathways, in which butyrate and/or hexanaote serves as a substrate in metabolic pathways of certain microbial communities.

After baseline measurements, including blood sampling, the participant consumed a liquid HFMM within 5 min. This HFMM contained: 125 g whole milk “Campina volle melk” (FrieslandCampina, The Netherlands), 50 g whipped cream “Campina slagroom” (FrieslandCampina, The Netherlands), 150 g whipped ice cream “Hertog slagroomijs” (Unilever, The Netherlands) and 15 g sugar (Royal Consun, The Netherlands). This supplied a total energy of 2.6 MJ consisting of 61 E% fat (35.5 E% saturated fat, 18.8 E% monounsaturated fat, and 1.7 E% polyunsaturated fat), 33E% carbohydrates and 6.3 E% protein. Based on the Nederlands Voedingsstoffenbestand (NEVO), a database with more detailed information on nutrient composition of specific food products, the amount of butyrate and hexanoate already present in the HFMM was estimated at approximately 1.3 and 0.7 grams, respectively. In this liquid HFMM, the four different concentrations butyrate and hexanoate-enriched oil were incorporated. The choice for the use of these doses were based on prior research with a daily dose of sodium butyrate between 600 mg and 4 g, which were all considered safe (39, 40, 64, 65). These doses were usually spread out over the day resulting in 100 mg (64) or 2 g of sodium butyrate supplementation each time (39, 65). Since no human studies have been performed with hexanoate, it was decided to take a total SCFA range between 650 mg and 2,000 mg, with a 2:1 ratio for butyrate:hexanoate, to prevent unfavorable side effects such as gastrointestinal complaints. Hence, the participant received either: a low (650 mg SCFA, 419 mg C4, 231 mg C6), medium (1,325 mg SCFA, 854 mg C4, 471 mg C6), or high amount (2,000 mg SCFA, 1,290 mg C4, 710 mg C6) of butyrate and hexanoate-enriched triglycerides (Akovita SCT), or a placebo (Fritex high oleic sunflower oil) with comparable percentage of saturated and unsaturated LCFA as the investigational products but no butyrate or hexanoate (Supplementary Tables 1, 2). Thus, on all four CIDs, participants received a serving of 10 g of the oil containing different amounts of SCFA.

To acquire a full picture on the effect of butyrate and hexanoate-enriched triglycerides on substrate metabolism, venous blood sampling was repeated at 30, 60, 120, 180, 240, 300, and 360 min after consumption of the HFMM containing the investigational products. VAS scores and breath analysis were assessed at the same time points and GSRS was determined every 2 h. After a few CIDs, we decided to take additional half hour time points after 180 min for the Gastrolyzer, to ensure we would not miss a peak in fermentation between hours. Participants were asked to maintain their habitual diet and physical activity level throughout the entire study period.

### 2.4. Biochemical analyses

Blood was collected in pre-chilled EDTA tubes (Becton Dickinson, NL) for the analysis of all metabolites, cytokines, and hormones. For SCFA analysis, blood was sampled in a pre-chilled 5 mL heparin tube (Becton Dickinson, NL) because EDTA interferes with the chromatography results. After collection, all tubes were centrifuged at 3,000 rpm, 4°C for 15 min, whereafter plasma was immediately aliquoted, snap-frozen in liquid nitrogen and stored in a −80°C freezer until analysis.

For SCFA and BCFA analysis, the plasma sample preparation procedure was done in accordance to Han et al. (66) with slight modifications. In brief, a portion of 150 μl plasma was used for SCFA and BCFA determination. An internal standard, 2-ethylbutyric acid, at a concentration of 36 μM was added. Derivatization was performed by adding 25 μl of a 160 mM 3-nitrophenylhydrazine (NPH) solution and 25 μl of a 200 mM 1-ethyl-3-(3-dimethylamino-propyl) carbodiimide hydrochloride solution. This mixture was incubated at 50°C for 30 min. Preparation of the standards curves was done in the same manner as the plasma samples. Concentration range of the standards was 5–400 μM for acetic acid and 0.1–20 μM for the other SCFA. The SCFA-NPH and BCFA-NPH derivatives were extracted by adding diethylether, mixed for 15 min and centrifuged at 6,000 g for 10 min. The upper diethylether layer was collected and dried under a flow of N_2_ gas. Finally, the residue, which contains the SCFA-NPH and BCFA-NPH derivatives, was dissolved in 60 μl methanol:water 25:75 (v/v) and directly measured on the liquid chromatography–mass spectrometry (LC-MS). This liquid chromatography was performed using a micro flow ultra-high-performance liquid chromatography (UHPLC) instrument (Dionex Ultimate 3000) at a flow rate of 200 μl/min 0.20 μl of the SCFA extract was loaded onto a Hypersil gold C18 UHPLC column, 10 cm x 2.1 mm inner diameter, 1.9 μm particle size (Thermo Fisher Scientific) at 35°C. The mobile phases consisted of solvent A (methanol:water:formic acid:ammonium formate, 10:90:0.1:0.05 v/v) and solvent B (methanol:water:formic acid:ammonium formate, 99:1:0.1:0.05 v/v). The following gradient conditions were applied: 0–2 min isocratic at 25% solvent B. From 2–11 min a linear gradient from 25 to 98% solvent B was applied, from 11 to 12.5 min isocratic at 98% solvent B to wash away any residual peaks eluting after last peak of interest and from 12.5 to 16 min isocratic at 25% solvent B to the initial gradient for re-equilibration of the column. The UHPLC was coupled on-line to a Q Exactive (Thermo Fisher Scientific) with heated electrospray ionization probe (Thermo Fisher Scientific). The mass spectrometer was operated in negative ionization mode. Measurements were done in a targeted selected ion monitoring mode. Resolution was set at 70.000 with a maximum injection time of 200 ms and an isolation window of 4 m/z. The inclusion list was 194.06 m/z for acetic acid-NPH, 208.07 m/z for propionic acid-NPH, 222.09 m/z for butyric acid-NPH and iso-butyric acid-NPH, 236.10 m/z for valeric acid-NPH, isovaleric acid-NPH and 2-methyl-butric acid-NPH, and 250.12 m/z for caproic acid-NPH. Quantification by integration of the peak areas and plotting each calibration curve was conducted by the workstation Thermo Scientific Xcalibur 4.4 software (Thermo Fisher Scientific).

To evaluate plasma FFA, triglycerides and glucose in the plasma samples, an automated spectrophotometer (ABX Pentra 400 autoanalyzer, Horiba ABX, Montpellier, France) and an automated Cobas Fara spectrophotometric auto-analyzer (Roche Diagnostics, Basel, Switzerland) were used. Circulating plasma insulin was assessed by commercially available radioimmunoassay (RIA) kits (Human Insulin specific RIA, Millipore Corporation, Billerica, MA, USA) and the concentration of inflammatory markers TNF-α, IFN-γ, IL-6, and IL-8 were evaluated by a multiplex enzyme-linked immuno-sorbent assay (Human ProInflammatory II 4-Plex Ultra-Sensitive Kit, Meso Scale Diagnositics, Rockville, MD, USA) and anti-inflammatory marker IL-10 was assessed by a human IL-10 Uncoated ELISA (Thermo Fisher Scientific) according to the manufacturers protocol. Samples were sent to Novo Nordisk Foundation Center for Basic Metabolic Research (University of Copenhagen, Copenhagen, Denmark) for GLP-1 analysis. Total GLP-1 immunoreactivity was evaluated as described previously (67) using antiserum, which reacts equally with the intact GLP-1 molecule and the primary (N-terminally truncated) metabolite of GLP-1.

### 2.5. Statistical analysis

Sample size was determined by a power calculation based on prior acute studies with colonic SCFA infusions (sodium acetate, sodium butyrate, sodium propionate) (52, 53) using GPower (Version 3.1 for Mac, Parkville, Victoria, Australia). In order to detect a 30 % increase (with a SD of 5%) in the primary outcome parameter between the different consumed concentrations of butyrate and hexanoate and reach a power of 95 % at a 2-sided α-level of 0.05, a total of 12 participants were required. Assuming an expected drop-out rate of 15 percent, 14 participants were included.

All data are presented as mean ± SEM. The incremental area under the curve (iAUC) was calculated by the trapezoid method to indicate relative changes compared to baseline. The postprandial response is expressed as iAUC and values are provided as a total (6 h) or split in periods of 2 h (0–2 h, 2–4 h, 4–6 h). The split in 2 h periods was done to assess changes in plasma SCFA levels and related metabolic effects in different time periods of the postprandial phase. Normality of the residuals was checked by histograms and goodness of fit was evaluated by computing QQ plots. Differences in postprandial iAUC between the four different concentrations of butyrate and hexanoate-enriched triglycerides were determined by using a linear mixed model as this model can account for correlations induced by repeated measurements performed on the same individual. Intervention and period (intervention order) were set as fixed factors and participants were set as random factor. *Post-hoc* analyses with pairwise comparisons were conducted only if a significant effect or a trend (*p* < 0.10) of the intervention was observed. Due to the explorative nature of these studies, least significant testing (LSD) was used for the *post-hoc* comparison. In-text described *p*-values refer to *post-hoc* comparisons and the *p*-value for overall treatment effect (Type III Test of Fixed Effects) can be found in the description of the corresponding figure. For the analysis of GSRS data (ordinal data), a non-parametric Friedmann test was used. SPSS 27.0 (Chicago, IL, USA) was used for all statistical analysis and a *p*-value < 0.05 (two-sided) was regarded statistically significant. A *p*-value between 0.05 and 0.10 was referred to as a trend.

## 3. Results

### 3.1. Esterifying butyrate and hexanoate-enriched triglycerides with LCFA delays the release of these SCFA in TIM-1

**Figure 2:**
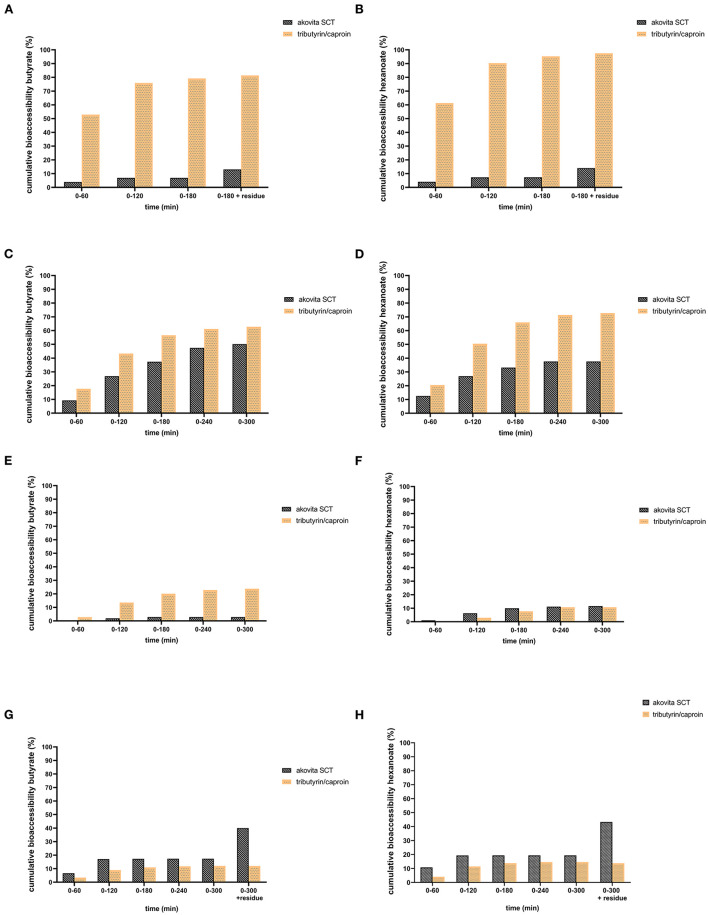
Comparison of cumulative bioaccessibility of butyrate **(A, C, E, G)** and hexanoate **(B, D, F, H)** from Akovita SCT or tributyrin/caproin in TIM-1 (as percentage of intake after correction for recovery). **(A, B)** Gastric compartment. **(C, D)** Jejunum compartment. **(E, F)** Ileal compartment. **(G, H)** Ileal efflux and residue. Akovita SCT = C4 and C6 fatty acids esterified with palm stearin and shea olein; containing 4.7% butyrate and 1.9% hexanoate. Tributyrin/caproin = C4 and C6 fatty acids linked to glycerol as a triglyceride; containing 71% butyrate and 29% hexanoate. The residue indicates the fraction that is left in the system after the 3 h (for gastric) or 5 h (for complete TIM-1)- experiment. From these results it appears more fatty acids are released in the stomach from tributyrin/caproin than from Akovita SCT **(A, B)**. Most of the tributyrin/caproin is digested after 300 min whereas with Akovita SCT, a large part remains in the ileal efflux and residue **(G, H)**.

In the TIM-1 experiments, a difference in bioaccessibility between Akovita SCT and tributyrin/caproin was observed. In the gastric compartment, supplementation with Akovita SCT resulted in a release of approximately 14% of the SCFA. Half of the released SCFA was delivered to the small intestine in its free form and the other 7% was collected in the residue after termination of the experiment. Hence, about 86% of the SCFA were still esterified to glycerol after passage through the gastric compartment and was delivered to the small intestine still as an intact (mono, di, or) triglyceride. In contrast, release of the SCFA from tributyrin/caproin in the gastric compartment was much higher and was close to or equalled the theoretical bioaccessibility (81% butyrate release and 98% hexanoate release, see Figures 2A, B). Consequently, the majority of the short-chain fatty acids were delivered to the small intestine compartment in free form.

In line with the low release of butyrate and hexanoate from Akovita SCT by the gastric lipase, approximately 50% of these acids were bioaccessible. The highest absorption occured in the jejunum (Figures 2C, D). Especially for butyrate, absorption in the ileum was negligible whereas this was slightly higher for hexanoate (Figures 2E, F). For tributyrin/caproin, practically all acids were delivered from the stomach already in free form, absorption was high, and reached between 80 and 90% of the intake. The kinetics of the absorption of butyrate and hexanoate were slightly different, with a higher absorption in the jejunal fraction for hexanoate than for butyrate. The ileal efflux together with the product that remained in the model (residue), provided an indication of the fraction of butyrate and hexanoate-enriched triglycerides, either as an intact triglyceride or (partly) broken down to FFA, mono-, or diglycerides, that would be delivered to the large intestine (Figures 2G, H). For Akovita SCT this was 40 and 43% for butyrate and hexanoate respectively, whereas this was 12 and 14% and tributyrin/caproin respectively, which is in line with the high absorption of tributyrin/caproin in the small intestine.

Nevertheless, this is only an approximation since part of the meal is still present in the TIM-system (due to its design, and due to the fact that we chose to terminate the experiment after ~80–85% of the meal has transited through the small intestine) after the 5-h experiment. After the termination of the experiment, the residues in all these compartments were collected and analyzed as well. Normally, these fractions will continue to be digested in the human individual. Additionally, to allow for determination of a mass balance, these fractions (and the residue) were saponified before analysis to release the acids from glycerol. Hence, it is unclear whether the acids that are present in the ileal efflux are present in free form or are still esterified as mono-, di-, or triglycerides. Exactly how much of this fraction present in the residue will still be absorbed in the last part of the small intestine and in the large intestine cannot be predicted on the basis of the current experiments.

Akovita SCT appeared to be released gradually and may reach the gastrointestinal tract more distally. In contrast, the release of SCFA from tributyrin/caproin is rapid and mainly takes place in the stomach, which is very comparable to the absorption of SCFA in its free form. Since we aimed for a product that was easy consumable and released more distally in the gastrointestinal tract, the *in vivo* study was conducted with Akovita SCT.

### 3.2. Butyrate and hexanoate-enriched triglycerides are well-tolerated *in vivo*

Between January 2021 and July 2021, fourteen participants were included in this study, of which twelve individuals completed all four CIDs. One participant dropped out after completion of the first CID and another participant dropped out before the start of the first CID (Supplementary Figure 2). Both participants discontinued for private reasons unrelated to the study. Hence, twelve individuals were analyzed for both primary and secondary outcomes. At screening, the included individuals had a mean age of 59 ± 2 years, a BMI of 29.5 ± 0.8 kg/m^2^ and were normoglycemic (fasting glucose 5.4 ± 0.1 mmol/L, HbA_1c_ 36.5 ± 1.1 mmol/L). No adverse events were reported during the study. An overview of participants' characteristics is displayed in Table 1. All four doses of butyrate and hexanoate-enriched triglycerides were well-tolerated and did not increase gastrointestinal complaints or discomfort, as determined by a GSRS, 2, 4, and 6 h after consumption of the liquid HFMM containing the test products (Supplementary Figure 3). One participant reported soft to watery stool ~4 h after consumption of the medium and the high dose, without any further symptoms.

**Table 1:** Participants' characteristics.

**Participants**	**Total (*n* = 12)**
Age (years)	59 ± 2
Weight (kg)	93.2 ± 3.4
Length (m)	1.78 ± 0.02
BMI (kg/m^2^)	29.5 ± 0.8
Hip circumference (cm)	104 ± 3
Waist circumference (cm)	105 ± 3
WHR	1.0 ± 0.02
SBP (mm/Hg)	137.2 ± 3.0
DBP (mm/Hg)	85.0 ± 2.3
Fglu (mmol/L)	5.4 ± 0.1
HbAc1 (mmol/L)	36.5 ± 1.1
HbAc1 (%)	5.5 ± 0.1
ALAT (U/L)	32 ± 3
Creatinine (μmol/L)	93 ± 5

ALAT, alanine aminotransferase; BMI, body mass index; DBP, diastolic blood pressure; Fglu, fasted plasma glucose; HbA1c, hemoglobin A1c; SBP, systolic blood pressure; WHR, waist-to-hip ratio. Values are given as means ± SEM.

### 3.3. Butyrate and hexanoate-enriched triglycerides increase butyrate and hexanoate in the circulation

**Figure 3:**
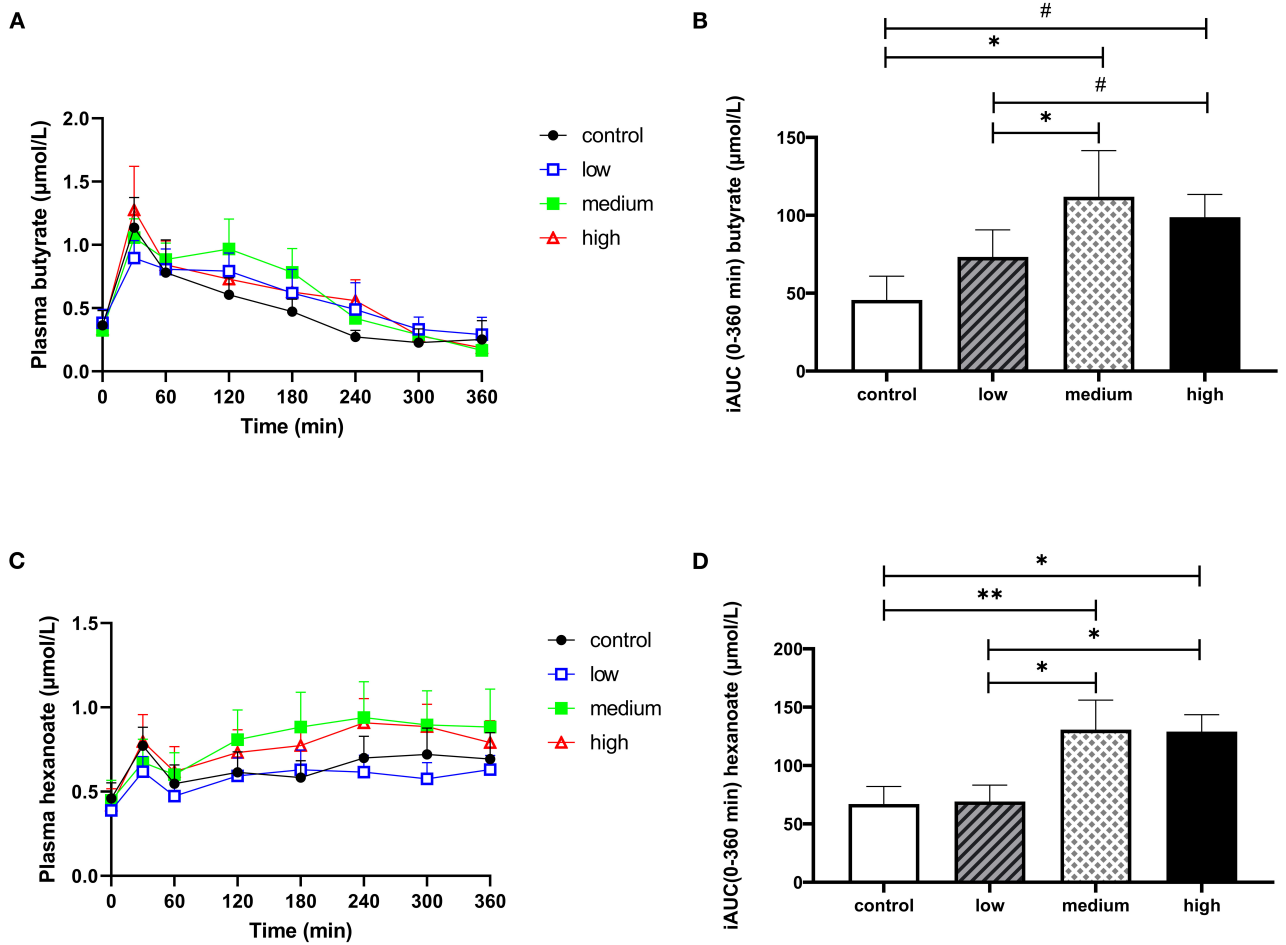
The effect of butyrate and hexanoate-enriched triglycerides on the circulating short chain fatty acids. **(A, B)** Butyrate and **(C, D)** hexanoate. **(A)** Postprandial plasma butyrate concentrations (t0–t360 min) after butyrate and hexanoate-enriched triglyceride ingestion. **(B)** iAUC for postprandial (t0–360 min) plasma butyrate following butyrate and hexanoate-enriched triglyceride ingestion. Overall treatment effect (Type III Test of Fixed Effects) for postprandial plasma butyrate *p* = 0.032. **(C)** Postprandial plasma hexanoate concentrations (t0–t360 min) after butyrate and hexanoate-enriched triglyceride ingestion. **(D)** iAUC for postprandial (t0–360 min) plasma hexanoate following butyrate and hexanoate-enriched triglyceride ingestion. Overall treatment effect (Type III Test of Fixed Effects) for postprandial plasma hexanoate *p* = 0.007. Values are presented as means ± SEMs (*n* = 12). Differences in postprandial iAUC between the four doses interventions (control, low, medium, high) were analyzed by a linear mixed model for repeated measures and the indicated *p*-values represent *post-hoc* testing. Statistical significance indicated with an asterisk (*) if *p* < 0.05, (**) if *p* < 0.01 and (#) if *p* < 0.10, indicating a trend. iAUC, incremental area under the curve.

Our primary aim was to determine whether orally administrated butyrate and hexanoate incorporated into triglycerides reach the circulation in men with overweight or obesity. Data on plasma SCFA are presented in Figure 3, Supplementary Table 3, and Supplementary Figures 4, 5. Baseline circulating SCFA were not significantly different between experimental groups (Supplementary Table 3). Consumption of the liquid HFMM alone (placebo) increased postprandial circulating butyrate and hexanoate, peaking at approximately 30 min after ingestion. The oil containing the medium concentration increased postprandial circulating butyrate compared to placebo (total iAUC_t0−*t*360*min*_, *p* = 0.026) and compared to the low dose (total iAUC_t0−*t*360*min*_, p = 0.027) (Figure 3A and Supplementary Table 3). The oil containing the high dose tended to increase postprandial circulating butyrate compared to placebo (total iAUC _t0−*t*360*min*_, *p* = 0.053) as well as compared to the low dose (total iAUC_t0−*t*360*min*_, *p* = 0.083) (Figure 3B and Supplementary Table 3). Similar patterns were observed for postprandial circulating hexanoate, in which both medium (total iAUC_t0−*t*360*min*_, *p* = 0.009) and high dose (total iAUC _t0−*t*360*min*_, *p* = 0.017) increased circulating levels compared to placebo as well as compared to low dose (*p* = 0.011 and *p* = 0.036 for medium and high, respectively) (Figures 3C, D and Supplementary Table 3). In contrast, the low dose did not alter circulating butyrate and hexanoate concentration compared to placebo. Although 10 out of the 12 participants displayed increased total iAUC for circulating butyrate and hexanoate after consumption of the oil containing either the medium or high dose (whereas two participant exhibited a slight reduction) compared to placebo, there was a considerable inter-individual variability in the magnitude of the response (Supplementary Figure 4). Surprisingly the participants that showed a decrease in iAUC were different individuals for the medium than for the high dose. The total iAUC_t0−*t*360*min*_ of other circulating SCFA including acetate, propionate, and valerate did not change significantly in response to the intervention (Supplementary Figures 5A–H and Supplementary Table 3).

### 3.4. Butyrate and hexanoate-enriched triglycerides ingestion may increase circulating branched fatty acids between 4 and 6 h after consumption

**Figure 4:**
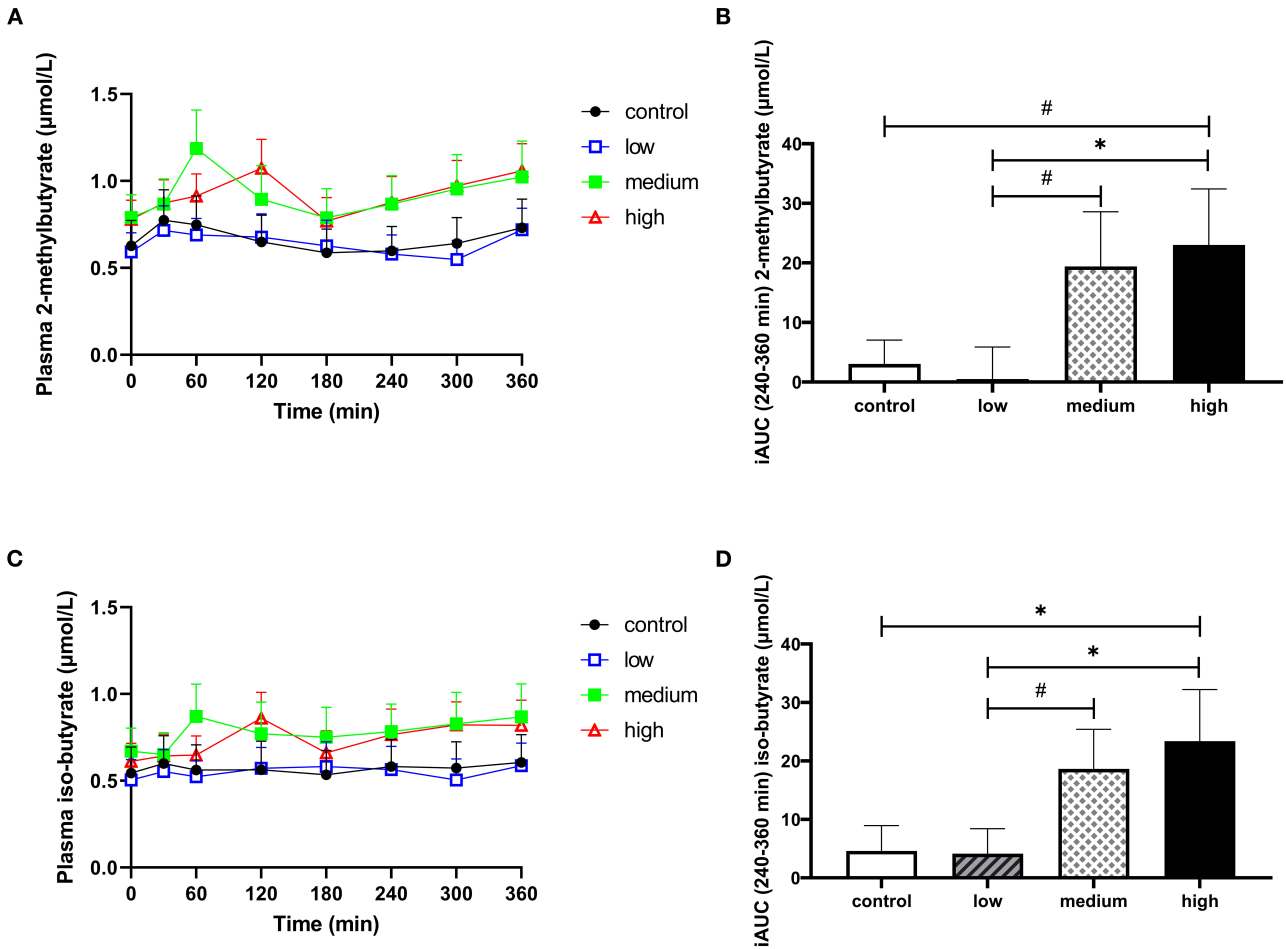
The effect of butyrate and hexanoate-enriched triglycerides on the circulating branched chain fatty acids. **(A, B)** 2-methylbutyrate and **(C,D)** iso-butyrate. **(A)** Postprandial plasma 2-methylbutyrate concentrations (t0–t360 min) after butyrate and hexanoate-enriched triglyceride ingestion. **(B)** iAUC for postprandial (t240–360 min) plasma 2-methylbutyrate following butyrate and hexanoate-enriched triglyceride ingestion. Overall treatment effect (Type III Test of Fixed Effects) for postprandial (t240–360 min) plasma 2-methylbutyrate *p* = 0.073. **(C)** Postprandial plasma iso-butyrate concentrations (t0–t360 min) after butyrate and hexanoate-enriched triglyceride ingestion. **(D)** iAUC for postprandial (t240–360 min) plasma iso-butyrate following butyrate and hexanoate-enriched triglyceride ingestion. Overall treatment effect (Type III Test of Fixed Effects) for postprandial (t240–360 min) plasma iso-butyrate *p* = 0.043. Values are presented as means ± SEMs (*n* = 12). Differences in postprandial iAUC between the four doses interventions (control, low, medium, high) were analyzed by a linear mixed model for repeated measures and the indicated p-values represent *post-hoc* testing. Statistical significance indicated with an asterisk (*) if *p* < 0.05 and (#) if *P* < 0.10, indicating a trend. iAUC, incremental area under the curve.

Data on plasma branched-chain fatty acids (BCFA) and hydrogen in breath are presented in Figure 4, Supplementary Table 3, and Supplementary Figure 5. There were no significant differences between interventions for total iAUC_t0−*t*360*min*_ for the BCFA 2-methylbutyrate, iso-butyrate, and iso-valerate. However, in the last 2 h of the CID (iAUC_t240−*t*360*min*_), plasma iso-butyrate significantly increased and plasma 2-methylbutyrate had a tendency to increase (Figures 4A–D). Supplementation of the high dose increased both 2-methylbutyrate and iso-butyrate concentration compared to placebo (iAUC_t240−*t*360*min*_, p = 0.066 & 0.033, respectively) and low dose (iAUC_t240−*t*360*min*_, *p* = 0.040 & 0.026, respectively). The medium dose tended to increase 2-methylbutyrate and iso-butyrate compared to the low dose (iAUC_t240−*t*360*min*_, *p* = 0.065 & 0.063, respectively). Iso-valerate did not change in response to intervention (Supplementary Table 3 and Supplementary Figures 5I, J). Hydrogen in breath was also not affected by butyrate and hexanoate-enriched triglyceride intake (total iAUC_t0−*t*360*min*_, *p* = 0.741, Supplementary Figures 5A, B).

### 3.5. Butyrate and hexanoate-enriched triglycerides ingestion does not directly affect subjective appetite, GLP-1 release, circulating metabolites, nor inflammatory markers

**Figure 5:**
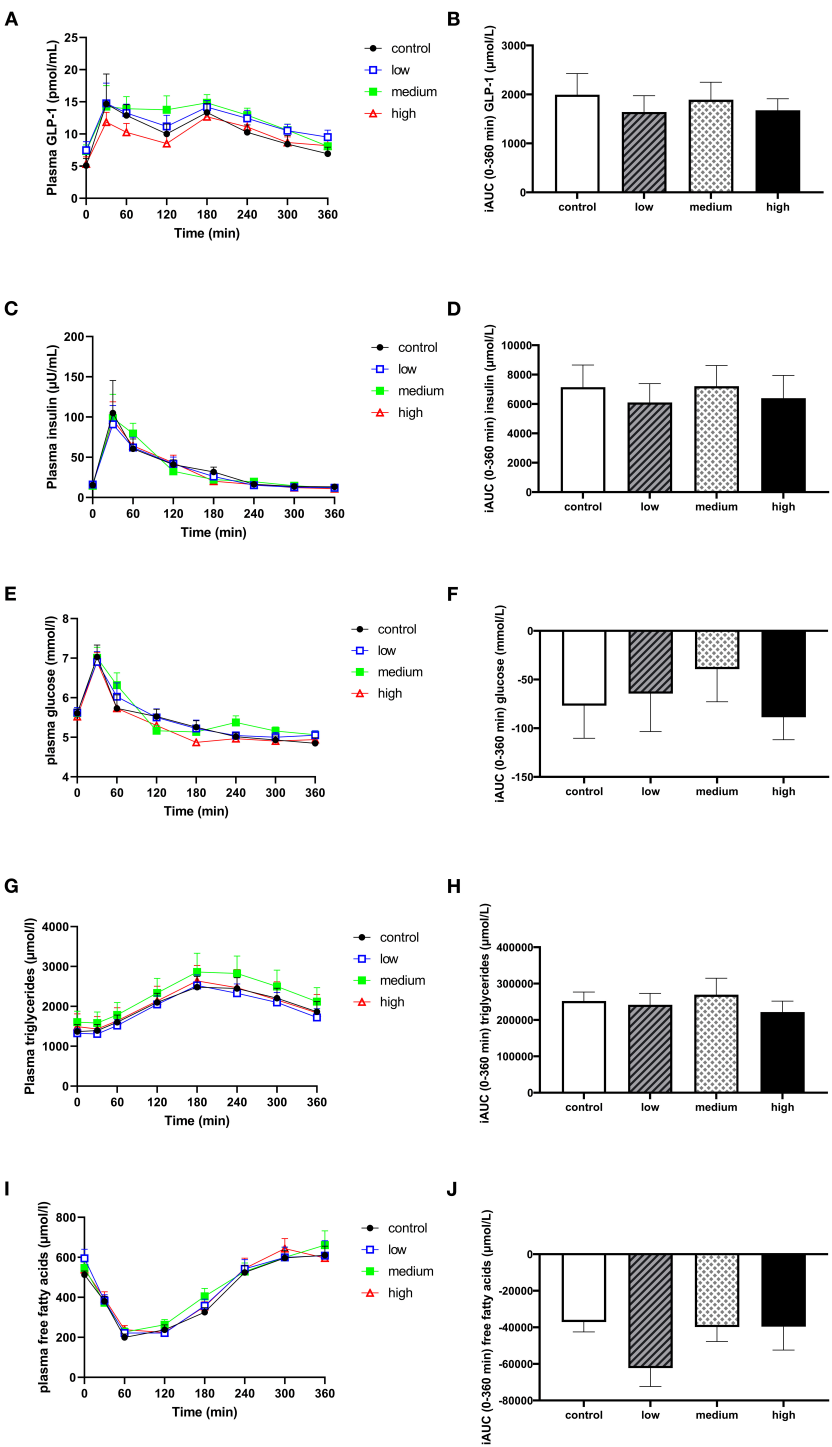
The effect of butyrate and hexanoate-enriched triglycerides on circulating GLP-1 **(A, B)** and metabolic parameters: insulin **(C, D)**, glucose **(E, F)**, triglycerides **(G, H)**, and free fatty acids **(I, J)**. **(A)** Postprandial plasma GLP-1 concentrations (t0–t360 min) after butyrate and hexanoate-enriched triglyceride ingestion. **(B)** iAUC for postprandial (t0–360 min) plasma GLP-1 following butyrate and hexanoate-enriched triglyceride ingestion. **(C)** Postprandial plasma insulin concentrations (t0–t360 min) after butyrate and hexanoate-enriched triglyceride ingestion. **(D)** iAUC for postprandial plasma insulin concentrations (t0-t360 min). **(E)** Postprandial plasma glucose concentrations (t0–t360 min) after butyrate and hexanoate-enriched triglyceride ingestion. **(F)** iAUC for postprandial plasma glucose concentrations (t0–t360 min). **(G)** Postprandial plasma triglyceride concentrations (t0–t360 min) after butyrate and hexanoate-enriched triglyceride ingestion. **(H)** iAUC for postprandial plasma triglyceride concentrations (t0–t360 min). **(I)** Postprandial plasma free fatty acid concentrations (t0–t360 min) after butyrate and hexanoate-enriched triglyceride ingestion. **(J)** iAUC for postprandial plasma free fatty acid concentrations (t0–t360 min). Values are presented as means ± SEMs (*n* = 12). Differences in postprandial iAUC between the four doses interventions (control, low, medium, high) were analyzed by a linear mixed model for repeated measures. iAUC, incremental area under the curve; GLP-1, glucagon-like peptide 1.

**Figure 6:**
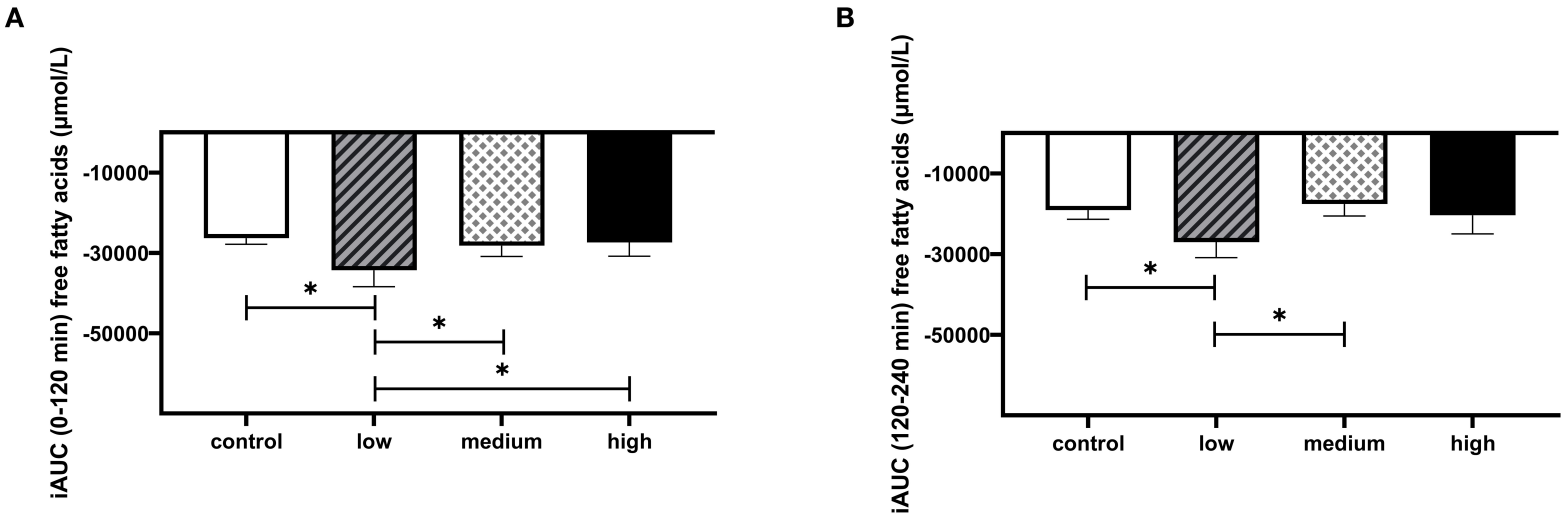
The effect of butyrate and hexanoate-enriched triglycerides on circulating free fatty acids in first 4 h after consumption. **(A)** iAUC for postprandial (t0–120 min) plasma free fatty acids following butyrate and hexanoate-enriched triglyceride ingestion. Overall treatment effect (Type III Test of Fixed Effects) for postprandial (0–2 h) plasma free fatty acids *P* = 0.086. **(B)** iAUC for postprandial (t120–240 min) plasma free fatty acids following butyrate and hexanoate-enriched triglyceride ingestion. Overall treatment effect (Type III Test of Fixed Effects) for postprandial (2–4 h) plasma free fatty acids P = 0.088. Values are presented as means ± SEMs (*n* = 12). Differences in postprandial iAUC between the four doses interventions (control, low, medium, high) were analyzed by a linear mixed model for repeated measures and the indicated p-values represent *post-hoc* testing. Statistical significance indicated with an asterisk (*) if *P* < 0.05. iAUC, incremental area under the curve.

There were no significant differences in postprandial circulating plasma GLP-1 (Figures 5A, B) or VAS scores for satiety and hunger (Supplementary Figure 6) between different doses. The consumption of butyrate and hexanoate-enriched triglycerides did not significantly affect postprandial insulin, glucose, triglycerides, and FFA patterns compared to control (Figures 5C–J). When looking at specific time slots, butyrate and hexanoate-enriched triglyceride consumption showed a tendency to affect circulating FFA for the first 2 h (0–2 h) and between 2 and 4 h (*p*-value < 0.10). However, only the low dose reduced circulating FFA (iAUC_t0−*t*120*min*_) compared to control (*p* = 0.018), medium dose (*p* = 0.043) and high dose (*p* = 0.048) (Figure 6A). Similar effects were observed for iAUC_t120−*t*240*min*_, with a significant reduction by the low dose compared to the control (*p* = 0.031) and medium dose (*p* = 0.021) (Figure 6B). Additionally, no overall treatment effect (Type III Test of Fixed Effects) was found on postprandial plasma concentrations of the cytokines: TNFα, IFNγ, Il-6, IL-8, and IL-10 (Supplementary Figure 7).

## 4. Discussion

*In vitro*, esterifying butyrate and hexanoate with LCFA delayed the release of these SCFA in the gastrointestinal tract compared to triglycerides consisting solely out of butyrate and hexanoate, which were almost completely hydrolyzed in the stomach. *In vivo*, oral intake of butyrate and hexanoate-enriched triglycerides consisting of 854 mg or 1,290 mg of butyrate combined with 231 and 710 mg of hexanoate, respectively, increased circulating postprandial butyrate and hexanoate compared to a LCFA sunflower oil control. The product was well-tolerated and did not lead to gastrointestinal complaints.

The increase in circulating butyrate and hexanoate after consumption of the medium and high dose is remarkable, since some evidence suggests that orally supplemented butyrate may not reach the circulation due to extensive splanchnic extraction (57, 58). Despite the significant increase in butyrate and hexanoate, we could not demonstrate a clear dose-response effect on circulating SCFA levels after consumption of the different doses of the butyrate and hexanoate-enriched triglycerides. This is most likely related to the small sample size, in which outliers may obscure a dose-response result. Additionally, in the current experimental design the placebo already contained a considerable amount of butyrate and hexanoate derived from the bovine milk fat present in the HFMM. Hence, the difference between control and low dose may be too small to allow significant changes in circulating SCFA. The similar potencies of the medium and high dose may also result from minor differences in the composition of triglyceride structures (Supplementary Table 4). The high dose, for instance, contains slightly more triglycerides consisting of 3 SCFA-esters whereas the medium dose contains more triglycerides with 2 LCFA- and 1 SCFA-ester. The performed TIM-1 experiments suggest this may affect the site and speed of triglyceride cleavage and, consequently, absorption of the SCFA. We also cannot exclude that with consumption of the medium dose a threshold is reached, where additionally ingested butyrate and hexanoate does not lead to more of these SCFA in the circulation.

Interestingly, the increase in circulating SCFA was accompanied by a trend toward increased circulating BCFA, especially iso-butyrate and 2-methylbutyrate, between 4 and 6 h after ingestion of the butyrate and hexanoate-enriched triglycerides. With LC-MS we confirmed BCFA were not present in the oil itself (Supplementary Figure 8). Although research still remains limited, some evidence suggests that the production of proteolytic metabolites including BCFA is associated with detrimental effects on host metabolism (68, 69). Not much data are available on the relationship between oral SCFA ingestion and circulating BCFA. Nonetheless, we speculate on a few potential hypotheses. The raise in BCFA is specifically observed four hours after the consumption of the oil and the TIM-1 results suggest ~40% of Akovita SCT still remains after 80–85% of transit in the small intestine is completed (either still attached to the glycerol backbone or as free SCFA). Possibly small quantities of butyrate and hexanoate may reach the microbiota in the large intestine and distal part of the small intestine and affect microbial fermentative processes here. Furthermore, butyrate is known to stimulate the production of serotonin in the intestinal tract and may hereby promote peristalsis and thereby shortening gut transit time (70–72). Faster transit though the small intestine may increase the level of undigested proteins entering the colon and, consequently increasing the availability of substrates for proteolytic fermentation (73). However, the increase in BCFA after SCFA-enriched triglycerides remains hard to explain and these hypotheses remain purely speculative.

Increased systemic availability of butyrate and hexanoate after ingestion of the medium and high dose may lead to these SCFA being taken up by metabolically active organs such as the liver, muscle, pancreas, and adipose tissue or bind to specific receptors at the surface of these tissues and alter processes involved in substrate and energy metabolism and inflammation (23). In contrast to our hypothesis, this increase in circulating butyrate and hexanoate concentrations did not translate into alterations in subjective appetite, hormones, and metabolic/inflammatory parameters in men with overweight/obesity in this acute setting of the study. The satietogenic effects of oral butyrate supplementation have been established in an obese mouse model (74) but, at present, have not been studied in well-controlled human clinical trials. Here we report no acute effect of butyrate and hexanoate-enriched triglycerides on subjective appetite, which could be potentially attributed to a lack of (extra) GLP-1 release. A clinical trial investigating acute rectal administration of SCFA mixtures, containing physiological amounts of butyrate (and acetate and propionate), also did not show an increase in fasting and postprandial circulating GLP-1 concentrations compared to placebo in men with overweight/obesity (53). In contrast, long-term (45 days) intervention with a low dose of oral sodium butyrate (600 mg/day) significantly increased fasting serum GLP- 1 concentrations in patients with T2DM compared to placebo (64). Prior work in rats suggested that mode of butyrate administration is important its effect on GLP-1, with a pronounced increase in circulating GLP-1 after increased cecal butyrate production induced by resistant starch supplementation but unchanged GLP-1 concentration after oral butyrate supplementation (75). Since the majority of the L-enteroendocrine cells that produce GLP-1 are located in the distal part of the small intestine (and colon) (76, 77), oral ingestion of butyrate and hexanoate-enriched triglycerides may not reach the distal small intestine in sufficient amounts to induce additional GLP-1 release. Altogether, the effect of butyrate on GLP-1 may vary for different modes of administration, distinct target populations and may depend on the extent of the intervention period (acute vs. chronic). The low dose of butyrate and hexanoate-enriched triglycerides showed a tendency to decrease circulating FFA in the first 4 h after consumption. In a human white adipocyte model, a low dose of butyrate (1 μmol/L) slightly increased lipolysis (78) and a high dose of hexanoate (0.5 mM) inhibited the activity of lipogenic genes in cultured chicken hepatocytes (79). Nevertheless, it seems unlikely that the SCFA in the low dose of oil reached the circulation and thereafter the adipose tissue in a significant amount to inhibit lipolysis.

The current study only included individuals that were overweight/obese and we did not include an additional group with healthy lean males. A lack of acute metabolic changes could signify that the tested concentrations of butyrate and hexanoate were insufficient to induce significant direct alterations in metabolism (which were pharmacologically relevant) in our specific target population. As mentioned previously, lean individuals were more sensitive to chronic butyrate intervention than individuals with metabolic syndrome, in which insulin sensitivity remained unchanged post sodium butyrate intervention (39). Nevertheless, metabolic and hormonal alterations may occur at a later stage than the 6 h that were assessed in this study. To illustrate, a rat study showed no acute insulinotropic effects after sodium butyrate supplementation, whereas prolonging sodium butyrate exposure to 24 h potentiated insulin secretion (80). Some of butyrate's beneficial effects on inflammation and glucose homeostasis have been attributed to HDAC inhibition (81–83). Such epigenetic modifications may require more time to develop. Several studies have shown elevated plasma butyrate concentrations in response to acute fiber supplementation or a fiber-rich meal in healthy individuals resulted in an improved postprandial glucose response the next day (54, 84–86). Thus, future studies could, for instance, investigate the effect of butyrate and hexanoate-enriched triglycerides on second meal response or evaluate whether changes in substrate metabolism occur after daily chronic supplementation (>3 months). Additionally, we cannot exclude that the absence of metabolic effects is caused by antagonism in which either butyrate or hexanoate obscures the beneficial effects of the other SCFA. To investigate this, the acute effect of both butyrate and hexanoate in humans should be studied separately.

Despite the evident strengths of this study by using a crossover design, as well as investigating different doses, this study has some limitations. From this study we cannot derive the quantity of the product that is recovered in the circulation and we cannot distinguish how much of the circulating SCFA is derived from the test product and how much from the HFMM itself. For this end, isotope-tracing studies can be used to identify the specific kinetics of triglyceride-derived SCFA and provide more specific information on the absorption, distribution, metabolism, and excretion of the SCFA. Although we studied the approximate composition of triglyceride structures among the different doses (percentage of triglycerides containing either 0, 1, 2, or 3 SCFA attached to the glycerol backbone), we do not know if the positioning of these SCFA varies among the different doses. Differences in stereospecific positioning of fatty acids in the triacylglycerol structure may influence the speed of cleavage by lipase and, subsequently, the location of SCFA release along the gastrointestinal tract (87). If butyrate is esterified at the α (sn-3) position (similar to butyric acid in bovine milk fat), this allows for rapid cleavage by gastric lipase (87, 88) whereas butyrate esterified at the β (sn-2) position is not cleaved by gastric lipase and only in minimal amounts by pancreatic lipase (89).

The increase in circulating butyrate and hexanoate appears gradual and persistent over several hours, which was in line with the release pattern observed in the TIM-1 experiments. A gradual release may be preferable over rapid large peaks in SCFA release that may coincide with sodium butyrate supplementation (and tributyrin/tricaproin). Additionally, although only a minor component of sodium butyrate, 4 g of sodium butyrate still results in an increased intake of ~850 mg of sodium, which is a substantial amount, especially considering individuals with overweight/obesity are at increased risk for hypertension and cardiovascular diseases (90). Lastly, incorporating butyrate into triglycerides greatly increases its product palatability, making it an easy-to-consume product for daily use. These triglycerides differ from those present in bovine milk fat both in positioning of the SCFA (can be positioned at the sn-1, 2, and 3 position vs. solely at the sn-3 position in bovine milk) and the amount of SCFA that can be attached to the glycerol backbone (1, 2, or 3 vs. solely 1 SCFA in bovine milk). Besides, to reach the amount of SCFA present in the medium or high dose of Akovita SCT, a considerable amount of full fat milk would have to be consumed (e.g., to reach the 1,290 mg of butyrate in the high dose one would have to ingest 1.2 liters of full fat milk). Collectively, these advantages and the observed effect on circulating SCFA offer great potential for the application of chronic SCFA-enriched triglyceride use in individuals with metabolic disturbances despite unaltered acute metabolic and inflammatory parameters. Especially, since several clinical studies have linked interventions resulting in increased circulating SCFA levels including butyrate to beneficial metabolic changes (53, 54).

To conclude this translational project, the *in vivo* study demonstrated for the first time that specific doses of butyrate and hexanoate-enriched triglycerides are able to acutely and gradually increase circulating butyrate and hexanoate in men with overweight/obesity. The *in vitro* experiments using TIM-1 specifically demonstrated butyrate and hexanoate-enriched triglycerides esterified with LCFA exhibit a delayed release of SCFA, which may explain this gradual increase in circulating SCFA. Long-term well-controlled randomized clinical trials should elucidate whether this steady and more chronic increase in circulating SCFA can be translated to beneficial metabolic changes and should determine whether this applies to males as well as females and to different metabolic phenotypes.

## Data availability statement

The datasets presented in this article are not readily available because the raw data supporting the conclusion will be made available upon reasonable request. Requests to access the datasets should be directed to emanuel.canfora@maastrichtuniversity.nl.

## Ethics statement

The studies involving human participants were reviewed and approved by Medical Ethics Committee of Maastricht University Medical Center+. The patients/participants provided their written informed consent to participate in this study.

## Author contributions

TD was responsible for the enrollment of participants, data collection, statistical analysis, and writing the original manuscript. EC supervision and provided guidance during clinical study, responsible for the final content of the manuscript, and received funding. EB, EC, KV, AH, and LS have discussed the content and reviewed and edited the manuscript. FB and JH have performed the laboratory analysis for determination of plasma SCFA and GLP-1, respectively. All authors have read and approved the submitted version of the manuscript.
