# Supplementary material for: Butyrate and hexanoate-enriched triglycerides increase postprandrial systemic butyrate and hexanoate in men with overweight/obesity: A double-blind placebo-controlled randomized crossover trial
Source: Front Nutr. 2023 Jan 4;9:1066950. doi: 10.3389/fnut.2022.1066950 (PMC9846253; doi:10.3389/fnut.2022.1066950)
Supplement: Supplementary file 1 [file Table_1.DOCX]

# **Supplementary material**

**1. Supplementary Figures and Tables**

**1.1 Supplementary figures**

Supplementary Figure 1. Schematic diagram of the dynamic, multi-compartmental model of the stomach and small intestine (TIM-1): A. stomach compartment; B. pyloric sphincter; C. duodenum compartment; D. peristaltic valve; E. jejunum compartment; F. peristaltic valve; G. ileum compartment; H. ileo-caecal sphincter; I. stomach secretion; J. duodenum secretion; K. jejunum/ileum secretion; L. pre-filter; M. semi-permeable membrane; N. water absorption; P. pH electrodes; Q. level sensors; R. temperature sensor. (1)


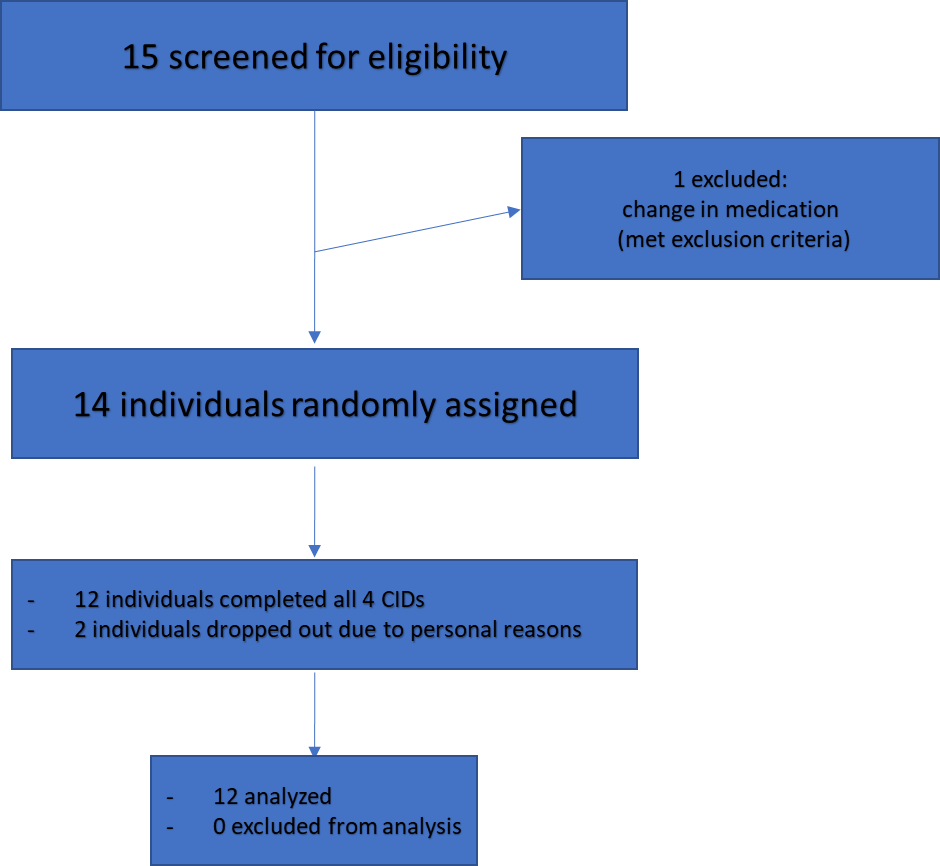


Supplementary Figure 2 - Flow chart of human participants included in this clinical study

Supplementary Figure 3. The effect of butyrate and hexanoate-enriched triglycerides on gastrointestinal complaints assessed by a Gastrointestinal Symptom Rating Scale (GSRS). Postprandial GSRS scores (t0-t360 min) indicated on a Likert scale after ingestion of butyrate and hexanoate-enriched triglycerides for: (A) abdominal pain, (B) abdominal discomfort, (C) bloating, (D) burping/regurgitation, (E) flatulence, and (F) nausea.

Supplementary Figure 4. Individual responses in circulating SCFA: butyrate (A, C) and hexanoate (B, D) for the medium dose (A,B) and high dose (C,D) butyrate and hexanoate-enriched triglycerides compared to control. Green indicates an increase in iAUC whereas red indicates a decrease in iAUC.

Supplementary Figure 5. The effect of butyrate and hexanoate-enriched triglycerides on general microbial activity and SCFA and BCFA production. Postprandial levels (t0-t360 min) after ingestion of butyrate and hexanoate-enriched triglycerides and corresponding iAUC of (A,B) hydrogen levels in breath as a measure of general microbial activity in the upper intestinal tract, and circulating SCFA: (C,D) acetate (E,F) propionate, and (G,H) valerate and the BCFA (I,J) isovalerate.

 Supplementary Figure 6. The effect of butyrate and hexanoate-enriched triglycerides on subjective feeling of hunger and satiety assessed by a Visual Analogue Scale (VAS). Postprandial VAS scores (t0-t360 min) after ingestion of butyrate and hexanoate-enriched triglycerides for (A) hunger, (B) fullness, (C) satiety, (D) thirst, (E) desire to eat. Participants draw a line between e.g. 0 = no feeling of hunger 10 = extreme feeling of hunger to indicate their subjective feeling of hunger.

Supplementary Figure 7. The effect of butyrate and hexanoate-enriched triglycerides on circulating inflammatory markers. (A,B) Postprandial plasma tumour necrosis factor α concentrations (t0-t360 min) and corresponding iAUC. (C,D) Postprandial plasma interferon γ concentrations (t0-t360 min) and corresponding iAUC. (E,F) Postprandial plasma interleukin 6 concentrations (t0-t360 min) and corresponding iAUC. (G,H) Postprandial plasma interleukin 8 concentrations (t0-t360 min) and corresponding iAUC. (I, J) Postprandial plasma interleukin 10 concentrations (t0-t360 min) and corresponding iAUC. Values are presented as means ± SEMs (n = 12). Abbreviations: iAUC, incremental area under the curve.

 Supplementary Figure 6. The effect of butyrate and hexanoate-enriched triglycerides on subjective feeling of hunger and satiety assessed by a Visual Analogue Scale (VAS). Postprandial VAS scores (t0-t360 min) after ingestion of butyrate and hexanoate-enriched triglycerides for (A) hunger, (B) fullness, (C) satiety, (D) thirst, (E) desire to eat. Participants draw a line between e.g. 0 = no feeling of hunger 10 = extreme feeling of hunger to indicate their subjective feeling of hunger.


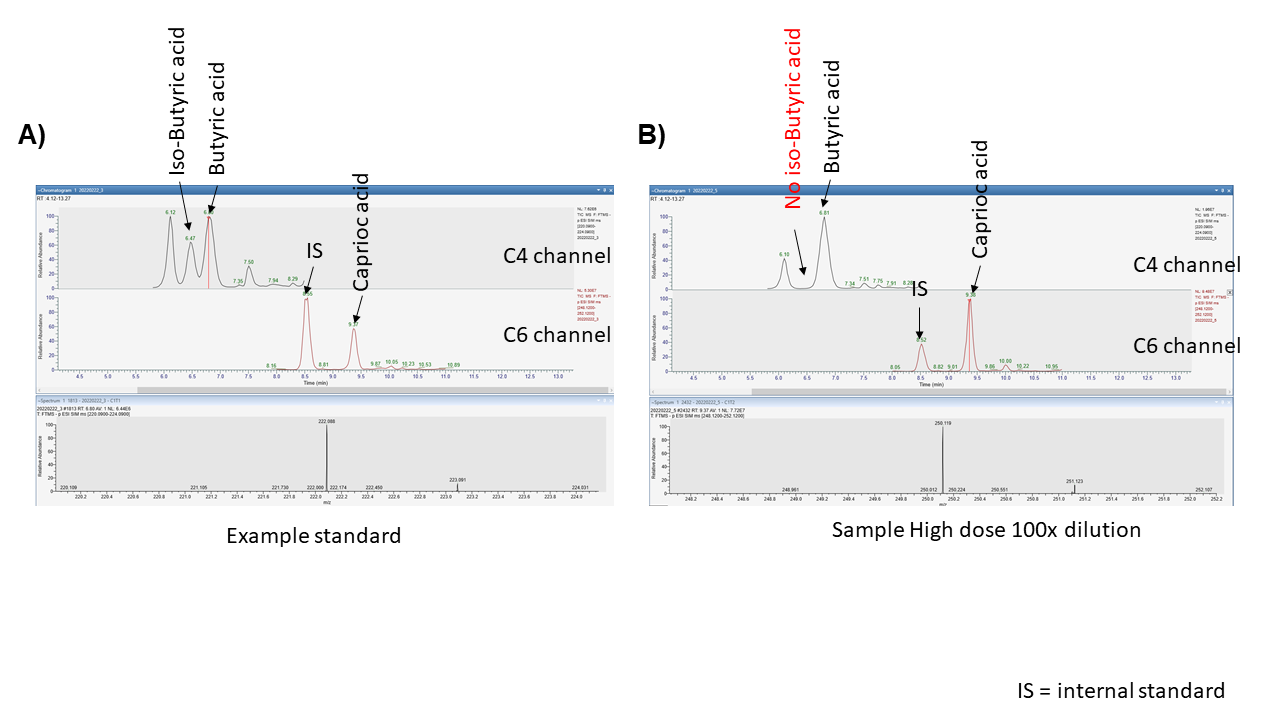


Supplementary Figure 8. (A) Example of LC-MS results of standard in which iso-butyrate peak is observed just before the peak of butyrate. (B) Example of LC-MS results of the oil containing the high dose of butyrate and hexanoate-enriched triglycerides in which there is no peak for iso-butyrate*.*

**1.2 Supplementary Tables**

Supplementary Table 1. Fatty acid composition of the intervention oils (g/100g fatty acids)

| Fatty acid | Low dose | | Medium dose | | High dose | | Control | |
| --- | --- | --- | --- | --- | --- | --- | --- | --- |
|  | Min | Max | Min | Max | Min | Max | Min | Max |
| SCFA (g) | 8.5 | - | 19.25 | - | 30 | - | - | 0 |
| C4:0 | 4.7 | - | 10.6 | - | 16.6 | - | - | 0 |
| C6:0 | 1.9 | - | 4.3 | - | 6.7 | - | - | 0 |
| Other fatty acid (g)s |  |  |  |  |  |  |  |  |
| C16:0 | 3 | 6 | 2.4 | 4.8 | 2.1 | 4.2 | 3 | 6 |
| C18:0 | 2.5 | 6 | 2 | 4.8 | 1.8 | 4.2 | 2.5 | 6 |
| C18:1 | 77 | 89.5 | 62.2 | 72.3 | 53.9 | 62.7 | 77 | 89.5 |
| C18:2 | 5 | 13 | 4 | 10.5 | 3.5 | 9.1 | 5 | 13 |

Abbreviations: C: carbon; Max: maximum; Min: minimum; SCFA: short chain fatty acids

Supplementary Table 2. Amount of fatty acids in the intervention oils per serving of 10 g

| Fatty acid | Low dose | | Medium dose | | High dose | | Control | |
| --- | --- | --- | --- | --- | --- | --- | --- | --- |
|  | Min | Max | Min | Max | Min | Max | Min | Max |
| SCFA (mg) | 650 | - | 1325 | - | 2000 | - | - | 0 |
| C4:0 | 419 | - | 854 | - | 1290 | - | - | 0 |
| C6:0 | 231 | - | 471 | - | 710 | - | - | 0 |
| Other fatty acids (g) |  |  |  |  |  |  |  |  |
| C16:0 | 0.3 | 0.5 | 0.2 | 0.5 | 0.2 | 0.5 | 0.3 | 0.6 |
| C18:0 | 0.2 | 0.5 | 0.2 | 0.5 | 0.2 | 0.5 | 0.2 | 0.6 |
| C18:1 | 6.8 | 8.0 | 6.3 | 7.4 | 5.8 | 6.8 | 6.8 | 8.6 |
| C18:2 | 0.4 | 1.2 | 0.4 | 1.1 | 0.4 | 1.0 | 1.0 | 1.2 |

Abbreviations: C: carbon; Max: maximum; Min: minimum; SCFA: short chain fatty acids

*Supplementary Table 3. Mixed model outcomes of postprandial circulating SCFA.*

| ***Variable*** | ***Period*** | **Mixed** | **Post hoc** | | | | |
| --- | --- | --- | --- | --- | --- | --- | --- |
|  |  | ***p-value, treatment*** | ***Placebo*** | ***Low*** | ***Medium*** | ***High*** | ***p-value*** |
| Plasma BA | *t*=0  μmol l^−1^ | 0.848 | 0.365 ± 0.1 | 0.386 ± 0.1 | 0.324 ± 0.07 | 0.335 ± 0.05 |  |
|  | iAUC_0–6_ _h_ μmol l^−1^per 6 h | **0.032** | 46 ± 15 | 73 ± 17 | 112 ± 30^a,b^ | 99 ± 15 | ^a^0.026, ^b^0.027, |
|  | iAUC_0–2_ _h_ μmol l^−1^per 2 h | **0.036** | 49 ± 9 | 47 ± 4 | 66 ± 12^b^ | 63 ± 13^b^ | ^b^0.009, ^b^0.029 |
|  | iAUC_2–4_ _h_ μmol l^−1^ per 2 h | 0.100 | 11 ± 7 | 29 ± 10 | 49 ± 16 | 36 ± 9 |  |
|  | iAUC_4–6_ _h_ μmol l^−1^per 2 h | *0.085* | -6 ± 8 | 11 ± 10 | 18 ± 11 | 21 ± 8^a^ | ^a^0.016 |
|  |  |  |  |  |  |  |  |
| Plasma HA | *t*=0  μmol l^−1^ | 0.960 | 0.459 ± 0.09 | 0.388 ± 0.08 | 0.448 ± 0.12 | 0.418 ± 0.10 |  |
|  | iAUC_0–6_ _h_ μmol l^−1^per 6 h | **0.007** | 67 ± 15 | 69 ± 14 | 131 ± 25^a,b^ | 129 ± 15^a,b^ | ^a^0.009, ^b^0.011, ^a^0.017, ^b^0.036 |
|  | iAUC_0–2_ _h_ μmol l^−1^per 2 h | *0.051* | 18 ± 3 | 17 ± 3 | 24 ± 5 | 30 ± 7^b^ | ^b^0.026 |
|  | iAUC_2–4_ _h_ μmol l^−1^ per 2 h | **0.007** | 19 ± 4 | 27 ± 8 | 52 ± 10^a,b^ | 45 ± 7^a^ | ^a^0.003, ^b^0.016, ^a^0.018 |
|  | iAUC_4–6_ _h_ μmol l^−1^per 2 h | *0.079* | 30 ± 11 | 25 ± 5 | 55 ± 13^b^ | 54 ± 9 | ^b^0.048 |
|  |  |  |  |  |  |  |  |
| Plasma AA | *t*=0  μmol l^−1^ | 0.438 | 39.6 ± 8.0 | 55.2 ± 7.7 | 55.7 ± 7.4 | 56.6± 17.2 |  |
|  | iAUC_0–6_ _h_ μmol l^−1^per 6 h | 0.445 | -1061 ± 1672 | -3922 ± 2114 | -5062 ± 2604 | -3558 ± 2831 |  |
|  | iAUC_0–2_ _h_ μmol l^−1^per 2 h | 0.258 | -1180 ± 444 | -2417 ± 562 | -2509 ± 649 | -2380 ± 881 |  |
|  | iAUC_2–4_ _h_ μmol l^−1^ per 2 h | 0.458 | -1143 ± 693 | -2143 ± 846 | -2506 ± 996 | -2100 ± 1075 |  |
|  | iAUC_4–6_ _h_ μmol l^−1^per 2 h | 0.642 | 1262 ± 786 | 638 ± 978 | -48 ± 1260 | 722 ± 1154 |  |
| Plasma PA | *t*=0  μmol l^−1^ | *0.059* | 1.63 ± 0.40 | 1.34 ± 0.21 | 1.65 ± 0.26 | 1.09 ± 0.14^a,c^ | ^a^0.015, ^c^0.026 |
|  | iAUC_0–6_ _h_ μmol l^−1^per 6 h | 0.421 | 68 ± 51 | 22 ± 15 | 99 ± 53 | 102 ± 43 |  |
|  | iAUC_0–2_ _h_ μmol l^−1^per 2 h | 0.248 | 27 ± 8 | 17 ± 7 | 31 ± 9 | 33 ± 11 |  |
|  | iAUC_2–4_ _h_ μmol l^−1^ per 2 h | 0.608 | 28 ± 20 | 12 ± 8 | 40 ± 23 | 39 ± 20 |  |
|  | iAUC_4–6_ _h_ μmol l^−1^per 2 h | 0.527 | 14 ± 30 | -7 ± 8 | 29 ± 26 | 31 ± 21 |  |
|  |  |  |  |  |  |  |  |
| Plasma | *t*=0  μmol l^−1^ | 0.116 | 4.121 ± 0.78 | 4.918 ± 0.82 | 6.149 ± 0.87 | 5.904 ± 0.94 |  |
| total VA | iAUC_0–6_ _h_ μmol l^−1^per 6 h | 0.521 | 95 ± 82 | 18 ± 74 | -88 ± 119 | -39 ± 133 |  |
|  | iAUC_0–2_ _h_ μmol l^−1^per 2 h | 0.926 | 93 ± 30 | 89 ± 35 | 93 ± 47 | 76 ± 30 |  |
|  | iAUC_2–4_ _h_ μmol l^−1^ per 2 h | 0567 | 11 ± 29 | -15 ± 32 | -62 ± 49 | -50 ± 58 |  |
|  | iAUC_4–6_ _h_ μmol l^−1^per 2 h | 0.255 | -9 ± 38 | -56 ± 38 | -119 ±47 | -64 ± 57 |  |
|  |  |  |  |  |  |  |  |
| Plasma | *t*=0  μmol l^−1^ | 0.457 | 0.627 ± 0.15 | 0.593 ± 0.11 | 0.789 ± 0.13 | 0.780 ± 0.11 |  |
| 2-MetBA | iAUC_0–6_ _h_ μmol l^−1^per 6 h | 0.234 | 11 ± 9 | 15 ± 10 | 48 ± 22 | 52 ± 26 |  |
|  | iAUC_0–2_ _h_ μmol l^−1^per 2 h | 0.926 | 11 ± 3 | 11 ± 3 | 23 ±7 | 18 ± 8 |  |
|  | iAUC_2–4_ _h_ μmol l^−1^ per 2 h | 0.539 | -3 ± 4 | 4 ± 6 | 5 ± 8 | 11 ± 11 |  |
|  | iAUC_4–6_ _h_ μmol l^−1^per 2 h | *0.073* | 3 ± 4 | 0.5 ±5 | 19 ± 9 | 23 ± 9^b^ | ^b^0.040 |
|  |  |  |  |  |  |  |  |
| Plasma | *t*=0  μmol l^−1^ | 0.751 | 0.684 ± 0.21 | 0.464 ± 0.10 | 0.615 ± 0.12 | 0.645 ± 0.10 |  |
| IsoVA | iAUC_0–6_ _h_ μmol l^−1^per 6 h | 0.323 | -41 ± 52 | 36 ± 7 | 44 ± 23 | 30 ± 17 |  |
|  | iAUC_0–2_ _h_ μmol l^−1^per 2 h | 0.436 | -5 ± 17 | 19 ± 3 | 19 ± 6 | 16 ± 7 |  |
|  | iAUC_2–4_ _h_ μmol l^−1^ per 2 h | 0.321 | -19 ±19 | 11 ± 4 | 8 ± 8 | 4 ± 7 |  |
|  | iAUC_4–6_ _h_ μmol l^−1^per 2 h | 0.281 | -17 ± 18 | 6 ± 3 | 17 ±10 | 11 ± 7 |  |
|  |  |  |  |  |  |  |  |
| Plasma | *t*=0  μmol l^−1^ | 0.691 | 0.544 ± 0.15 | 0.504 ± 0.12 | 0.671 ± 0.13 | 0.612 ± 0.10 |  |
| IsoBA | iAUC_0–6_ _h_ μmol l^−1^per 6 h | 0.146 | 9 ± 8 | 17 ± 18 | 41 ± 23 | 48 ± 8 |  |
|  | iAUC_0–2_ _h_ μmol l^−1^per 2 h | 0.285 | 3 ± 2 | 4 ± 5 | 11 ± 6 | 10 ± 2 |  |
|  | iAUC_2–4_ _h_ μmol l^−1^ per 2 h | 0.417 | 1 ± 2 | 8 ± 7 | 11 ± 10 | 15 ± 3 |  |
|  | iAUC_4–6_ _h_ μmol l^−1^per 2 h | **0.043** | 5 ± 4 | 4 ± 7 | 19 ± 9 | 23 ± 3^a,b^ | ^a^0.033, ^b^0.026 |

Abbreviations: 2-MetVA, 2-methylvalerate; AA, acetate; AUC, area under the curve; BA, butyrate; HA, hexanoate; iAUC, incremental area under the curve; IsoBA, iso-butyrate; IsoVA, isovalerate; PA. propionate; VA, valerate. Statistical analysis of selected variables by a mixed model for repeated measures and post hoc testing. Significant p-values for overall treatment effect (P<0.05) is indicated in bold and a tendency (0.05-0.10) is indicated in italic. ^a^Statistically significant from placebo. ^b^Statistically significant from low dose. ^c^Statistically significant from medium dose.

*Supplementary Table 4. Differences in composition of triglyceride structures between low, medium, and high dose*

| **Composition of triglyceride structures**  **(% of 10 grams of product)** | **low dose** | **medium dose** | **high dose** |
| --- | --- | --- | --- |
| Glycerol with 3 SCFA | 0 | 3.1 | 5.6 |
| Glycerol with 2 SCFA 1 LCFA | 6.8 | 18.7 | 29.2 |
| Glycerol with 1 SCFA, 2 LCFA | 31.2 | 38.8 | 29.9 |
| Glycerol with 3 LCFA | 57.1 | 36.6 | 34.7 |

Abbreviations: LCFA: long chain fatty acid consisting of either palm stearin (C16) and shea olein (C18); SCFA: short chain fatty acid consisting of either butyrate (C4) and/or hexanoate (C6).

# **References**

1. Keller D, Verbruggen S, Cash H, Farmer S, Venema K. Spores of Bacillus coagulans GBI-30, 6086 show high germination, survival and enzyme activity in a dynamic, computer-controlled in vitro model of the gastrointestinal tract. Benef Microbes. 2019;10(1):77-87.
